# Low Gilbert damping and high perpendicular magnetic anisotropy in an Ir-coupled L1_0_-FePd-based synthetic antiferromagnet

**DOI:** 10.1038/s41598-024-63475-0

**Published:** 2024-06-10

**Authors:** William K. Peria, Michael B. Katz, Jian-Ping Wang, Paul A. Crowell, Daniel B. Gopman

**Affiliations:** 1 School of Physics and Astronomy, University of Minnesota, Minneapolis, MN 55455 USA; 2 Materials Science and Engineering Division, NIST, Gaithersburg, MD 20899 USA; 3 Department of Electrical and Computer Engineering, University of Minnesota, Minneapolis, MN 55455 USA

**Keywords:** Magnetic properties and materials, Information storage

## Abstract

Thin ferromagnetic films possessing perpendicular magnetic anisotropy derived from the crystal lattice can deliver the requisite magnetocrystalline anisotropy density for thermally stable magnetic memory and logic devices at the single-digit-nm lateral size. Here, we demonstrate that an epitaxial synthetic antiferromagnet can be formed from L1_0_ FePd, a candidate material with large magnetocrystalline anisotropy energy, through insertion of an ultrathin Ir spacer. Tuning of the Ir spacer thickness leads to synthetic antiferromagnetically coupled FePd layers, with an interlayer exchange field upwards of 0.6 T combined with a perpendicular magnetic anisotropy energy of 0.95 MJ/m^3^ and a low Gilbert damping of 0.01. Temperature-dependent ferromagnetic resonance measurements show that the Gilbert damping is mostly insensitive to temperature over a range of 20 K up to 300 K. In FePd|Ir|FePd trilayers with lower interlayer exchange coupling, optic and acoustic dynamic ferromagnetic resonance modes are explored as a function of temperature. The ability to engineer low damping and large interlayer exchange coupling in FePd|Ir|FePd synthetic antiferromagnets with high perpendicular magnetic anisotropy could prove useful for high performance spintronic devices.

## Introduction

Ultrathin magnetic films with perpendicular magnetic anisotropy (PMA) are of significant technological interest for emerging magnetic random access memory (MRAM) devices to be applied in smart devices/wearables, enterprise storage and embedded memory^1–4^. The potential for high density and low write energy of magnetic tunnel junction (MTJ) devices (the key building block of MRAM) switched by spin-transfer-torque mainly depends on the magnetic properties of the ferromagnetic layers, such as the PMA value (*K*_u_), Gilbert damping (*α*), and thermal stability (*Δ* = *K*_u_*V*/*k*_B_*T*, where *V* is the volume of the free layer in MTJs, *k*_B_ is Boltzmann’s constant and *T* is ambient temperature)^5–9^. For the demand of the sub-10 nm node MTJ, a large *K*_u_ ($\approx$ MJ/m^3^) and small *α* values are required to maintain 10-year data storage and low switching current density for next-generation spin memory and logic devices. With the well-known interfacial PMA materials (e.g. Ta/CoFeB/MgO), relatively low *K*_u_ and high *α*^10^ make it challenging to satisfy these requirements^11,12^.

Recently, L1_0_ FePd exhibiting large *K*_u_ ($\approx$ MJ/m^3^) and low *α* (< 0.01) has attracted considerable interest for spin memory device applications^13–21^. We have demonstrated that the magnetic properties of FePd thin films can be tuned through various growth conditions, including the substrate inclination relative to an FePd magnetron sputter deposition source^22^. We additionally have demonstrated that FePd can be grown using a wide range of buffer layer materials, including Pt, Ru, Rh and Ir^23^.

The latter three of these Pt-group elements (Ru, Rh and Ir) additionally are known to deliver a substantial interlayer exchange coupling energy between ferromagnetic layers. The interlayer exchange coupling (IEC) across the non-magnetic layer in a ferromagnet(FM)/non-magnet(NM)/ferromagnet trilayer shows an oscillatory and decaying behavior with the NM thickness that can be ferromagnetic or anti-ferromagnetic^24–27^. However, the anti-ferromagnetic behavior—what is referred to as a synthetic antiferromagnet (SAF)—is of particular technological interest to spintronic applications as the building block of a fringe-field free reference layer for ultrahigh density spin-transfer-torque magnetic random access memory (STT-MRAM) cells^11,12^. Additionally, the SAF shows technological merit as the free layer for SAF magnetic tunnel junctions (MTJs)^20,28,29^ and as domain wall and Skyrmion bubble devices^30,31^, in which case the IEC torques can generate faster magnetization switching, enable high domain wall velocities and stabilize Skyrmions at elevated temperature compared to ferromagnetic counterparts.

Our previous results highlight the favorable epitaxial relationship between an epitaxial Ir buffer layer grown above a single-crystalline MgO(001) substrate and an FePd thin film: Ir(001) < 100 >||FePd(001) < 100 > due to the small lattice mismatch between the in-plane lattice of FePd (*a* = 0.38 nm, *c* = 0.37 nm) and the lattice spacing of Ir (*a* = 0.38 nm). Ir layers have become the cornerstone of perpendicularly magnetized synthetic anti-ferromagnets due to their combined large perpendicular magnetic anisotropy and interlayer exchange coupling energy^32,33^. Typically grown as a face-centered-cubic (111) textured film and as part of polycrystalline (111) magnetic multilayers including Co and Pt layers, these films exhibit high Gilbert damping and as a consequence have restrictively broad lineshapes for ferromagnetic resonance exploration. In this work, we present epitaxial FePd|Ir|FePd SAF trilayers with (001) < 100 > orientation. These samples have advantageously lower Gilbert damping than their counterparts coupled with Co/Pt multilayers and permit observation of both acoustic and optic resonance modes of the anti-ferromagnetically coupled FePd layers. With a combination of $\approx$ 1 MJ/m^3^ anisotropy energy, interlayer exchange energy of 2 mJ/m^2^ and Gilbert damping below 0.01, epitaxial FePd|Ir|FePd SAFs have significant potential for emerging magnetic memory technologies^23,34,35^.

## Results

### Perpendicular magnetic anisotropy and interlayer exchange in FePd|Ir|FePd synthetic antiferromagnets

FePd|Ir|FePd perpendicular SAFs (pSAFs) were produced for this study on Cr(15 nm)/Pt(8 nm) films grown on MgO(001) single-crystalline substrates. This buffer layer stacking structure delivers the best combination of high perpendicular magnetic anisotropy and low Gilbert damping for a single FePd layer^23,36^. We grew three FePd SAFs for this study: SAF A, FePd(4 nm)|Ir(1.3 nm)|FePd(5 nm); SAF B, FePd(4 nm)|Ir(0.9 nm)|FePd(5 nm) and SAF C, FePd(4 nm)|Ir(0.8 nm)|FePd(2.5 nm) (described in Methods section). Layer thicknesses were confirmed with cross-section transmission electron microscopy, detailed in the Supplementary Information section. The progression along this sample series is in the direction of increasing interlayer exchange field and implements SAFs with an Ir thickness *t*_Ir_ = 0.8 nm and 1.3 nm, which are near the positions of the first- and second anti-ferromagnetic coupling peaks of Ir, respectively. The first interlayer exchange coupling peak energy ${J}_{ex}$ conveys a nearly 2.6 mJ/m^2^ anti-ferromagnetic coupling and the second peak is two-to-three times lower^32,33^. Owing to the interfacial nature of this coupling through the Ir spacer, the interlayer exchange coupling field ${H}_{ex}$ decreases with increasing thickness *t* of the individual ferromagnetic layers: ${\mu }_{0}{H}_{ex}={J}_{ex}/{M}_{s}t$, where ${M}_{s}$ is the saturation magnetization of the ferromagnetic layer.

**Figure 1:**
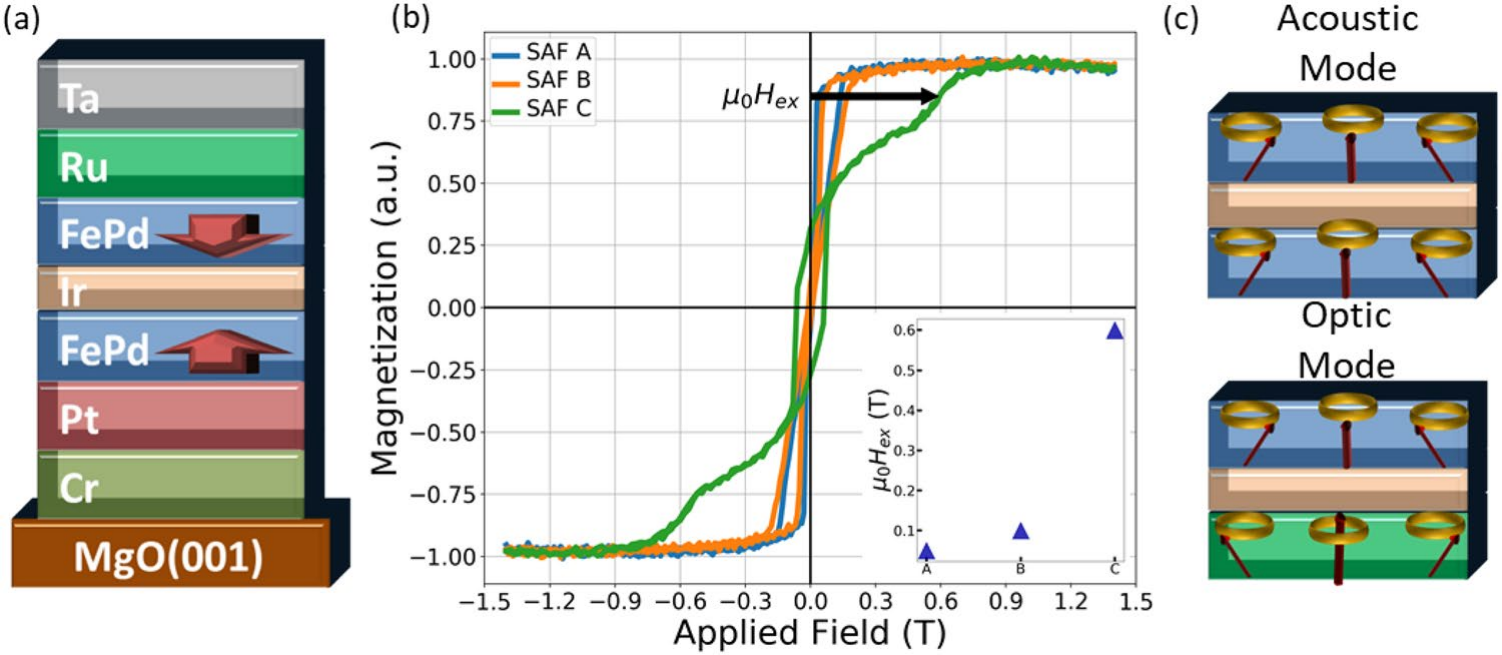
(**a**) Schematic Layer Stacking Structure of FePd Synthetic Antiferromagnet (SAF); (**b**) Out-of-plane Magnetic Hysteresis Curves for SAFs A, B and C; (**c**) Cartoon Depiction of Acoustic and Optic Modes Probed by Broadband Ferromagnetic Resonance Spectroscopy.

Figure 1 highlights the magnetic behavior of the FePd SAF structure. The layer stacking structure in Fig. 1a highlights the net-zero magnetization orientation of the two FePd layers at zero external field. The out-of-plane magnetization versus applied field hysteresis loops are shown in Fig. 1b. In progression along the SAF series, there is an increase in the hysteresis loop offset associated with the interlayer exchange field ${H}_{ex}$, proceeding from 0.06 T to 0.15 T to 0.6 T for SAFs A, B and C, respectively. This interlayer exchange field ${H}_{ex}$, is defined for SAFs A and B, as the offset of the center of the hysteresis loops, whereas for SAF C it is defined as the field at the midpoint of the reversible (no hysteresis) SAF rotation feature. While SAFs A and B exhibit sharp hysteretic reversal around their respective exchange fields, the gradual and reversible feature seen in SAF C signifies magnetization rotation of the layers from an anti-parallel to a parallel relative orientation. This transition takes place as the magnitude of the perpendicular magnetic anisotropy energy *K*_*u*_ approaches parity with the ratio of the interlayer exchange energy divided by the layer thickness ${J}_{ex}/t$.

In addition to the coupling of static magnetization of the two FePd layers in the pSAF, there is dynamic coupling of the layers into an in-phase (acoustic) mode and a 180° degree out-of-phase (optic) mode. These additional modes are depicted in Fig. 1c and their relative eigenfrequencies are separated by a quantity proportional to the interlayer exchange field. The higher frequency dynamics associated with the optic mode precession has generated interest for faster writing in magnetic memory devices^28,37–39^. However, the anti-ferromagnetic coupling generates an additional torque as a dynamical exchange between the layers that results in significant additional damping, making practical observation of the optic mode in a stripline ferromagnetic resonance absorption measurement difficult^40–42^. In the below, we will report on the optic mode only in the SAF A sample, as the other specimens did not exhibit an optic mode above the resolution of our spectrometer.

**Figure 2:**
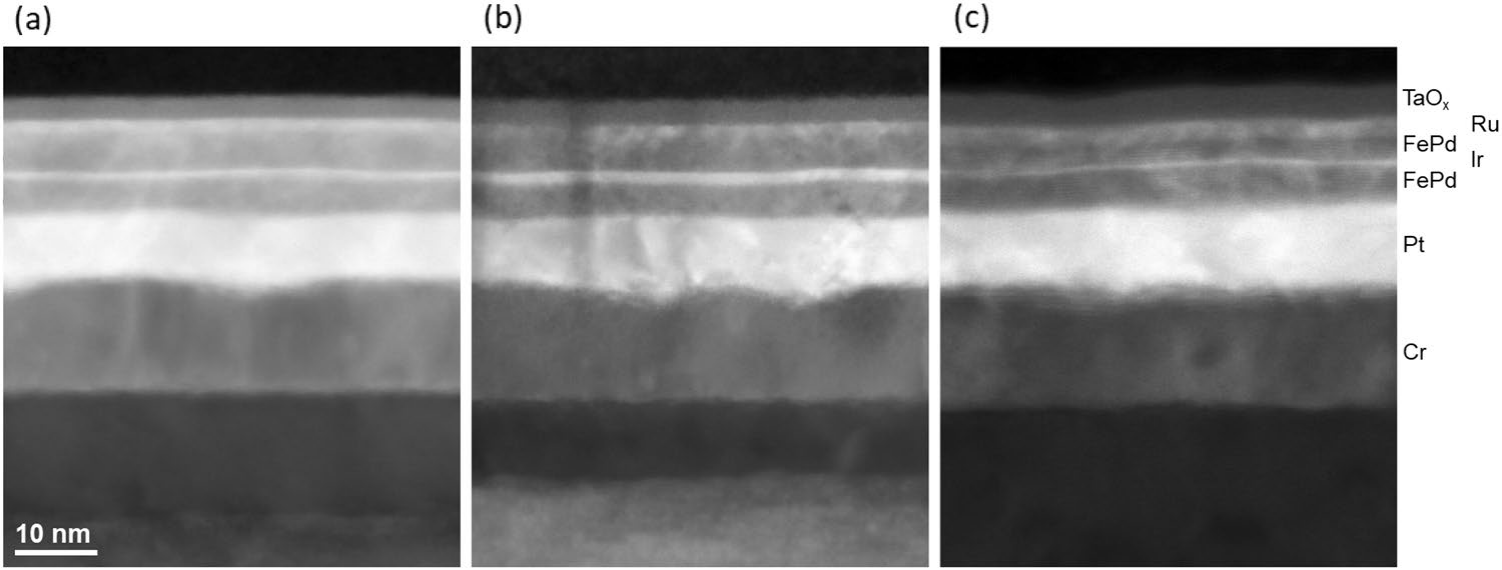
High-angle annular dark field scanning transmission electron micrographs of: (**a**) low-IEC SAF; (**b**) intermediate-IEC sample and (**c**) high-IEC SAF.

Controlled crystallizations and sharp interfaces are key to the perpendicular magnetization and controllable IEC field in FePd pSAFs. Symmetric x-ray diffraction (see Supplementary Fig. S1) reveals (001) texturing of each sample, and the presence of the (001) FePd peak allows estimation of the *L*1_0_ order parameter, ranging between 0.54 and 0.64 for the three samples. Figure 2 shows high-angle annular dark field scanning transmission electron micrographs (HAADF-STEM) of the three SAF samples. The precise control of the Ir spacer thickness is confirmed in Fig. 2a–c. Furthermore, all samples show sharp interfaces between the layers, indicating good chemical control of the interfaces even under the 500 °C post-annealing condition. While there is some long-wavelength undulation at the Cr/Pt interface, potentially associated with the Cr grain structure, we find that the upper Pt interface with FePd is flat across the three samples, as are the interfaces between FePd and Ir within the three SAF structures. The Supplementary Information section additionally details intensity depth profiling, in which an oscillatory contrast can be seen within the FePd layers for each sample, associated with the double contrast originating from *Z* contrast within the ordered Fe and Pd planes.

### Temperature dependence of the optic and acoustic modes of a low IEC pSAF

**Figure 3:**
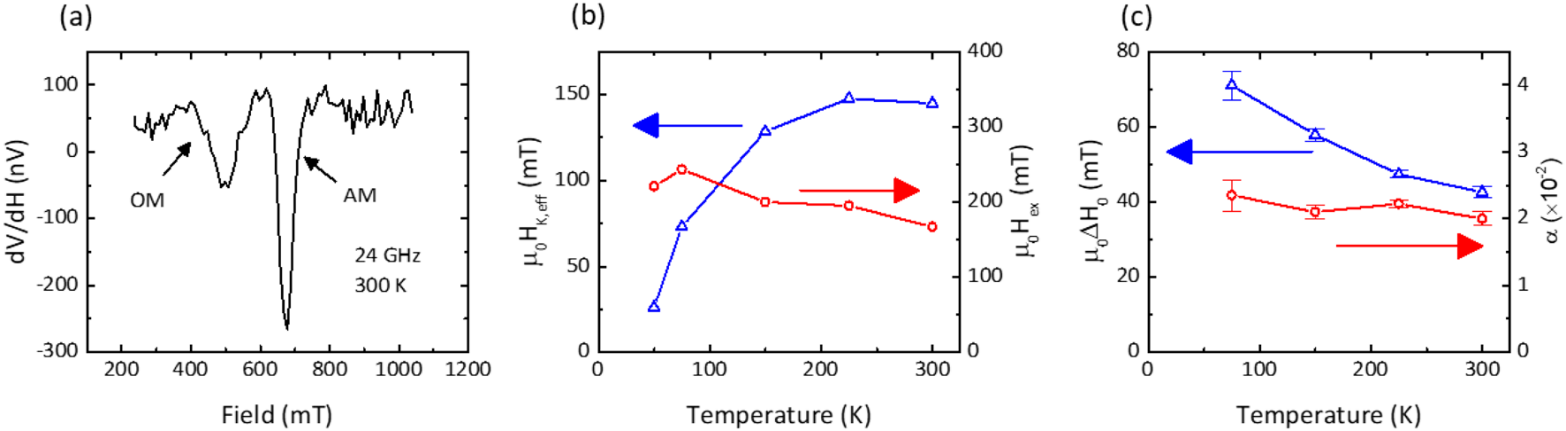
(**a**) Example lineshape of SAF A showing optic and acoustic resonance modes at *f* = 24 GHz; (**b**) temperature dependence of the effective perpendicular anisotropy and interlayer exchange coupling; and (**c**) temperature dependence of the Gilbert damping and inhomogeneous line broadening.

To examine the dynamic properties of FePd SAFs, we employ a broadband ferromagnetic resonance technique in a custom microwave system placed in a cryostat for temperature dependent measurements under applied magnetic fields perpendicular to the plane up to 2 T and excitation frequencies up to 40 GHz. Figure 3a shows an example of ferromagnetic resonance measurement of SAF A at room temperature and at a fixed excitation frequency of 24 GHz. At this frequency, we observe a larger amplitude resonance lineshape at 0.68 T and a smaller amplitude resonance lineshape around 0.52 T. The low and broad lineshape of the feature at lower field is associated with the higher energy optic mode, while the mode at 0.68 T is associated with the in-phase (acoustic) precession of the two FePd layers. At a series of fixed frequencies, we extract the resonance field and resonance linewidth for the optic and acoustic modes, respectively. We fit the resonance frequency (*f*) vs out-of-plane resonance field (*H*) to the linear Kittel dispersion appropriate for a uniaxial magnetic thin film in an applied perpendicular field:

$$f= {\gamma \mu }_{0}\left(H+{H}_{K,eff}+{H}_{ex}\right),$$

where $\gamma =g{\mu }_{B}/h$ is the gyromagnetic ratio, where *g* is the spectroscopic splitting factor, ${\mu }_{B}$ is the Bohr magneton and *h* is Planck’s constant, ${H}_{K,eff}=2{K}_{u}/{\mu }_{0}{M}_{s}-{M}_{s}$ is the effective perpendicular magnetic anisotropy^43^, ${\mu }_{0}$ is the vacuum permeability and the optic mode is offset by the interlayer exchange field ${H}_{ex}$.

Evaluation of the effective perpendicular anisotropy, interlayer exchange field and the spectroscopic *g*-factor is carried out through measurement of the frequency vs resonance field dispersion at temperatures ranging from 50 K up to 300 K, shown in Fig. 3b. While the *g*-factor is mostly flat from 300 K down to 75 K, a modest (2%) rise in the *g*-factor is seen at 50 K. On the other hand, there is a gradual reduction of the effective anisotropy from + 0.15 T at 300 K down to 0.025 T at 50 K. This is also accompanied by an increase in the interlayer exchange field from 0.17 T at 300 K up to 0.22 T at 10 K, with an apparent maximum value of 0.24 T at 75 K. This would appear to indicate a low-temperature saturation of the interlayer exchange strength, consistent with previous examinations of exchange coupled multilayers of Fe and Cr^44^.

Additionally, the resonance linewidth versus frequency is fit to the below linear relationship to estimate the Gilbert damping and the inhomogeneous linewidth broadening (ILB):

$$\Delta H=\frac{2\alpha f}{{\mu }_{0}\gamma }+\Delta {H}_{0},$$

where $\alpha$ is the phenomenological Gilbert damping and $\Delta {H}_{0}$ is the ILB. Figure 3c highlights the temperature dependence of the Gilbert damping of the acoustic mode and the ILB. The room temperature Gilbert damping (0.021) is higher than single-layer FePd films buffered by Cr/Pt ($\lesssim$ 0.01) and likely reflects the significant spin pumping induced by the 1.3 nm thick Ir spacer^23^.

### Temperature dependence of the Gilbert damping and perpendicular anisotropy of a moderate IEC pSAF

**Figure 4:**
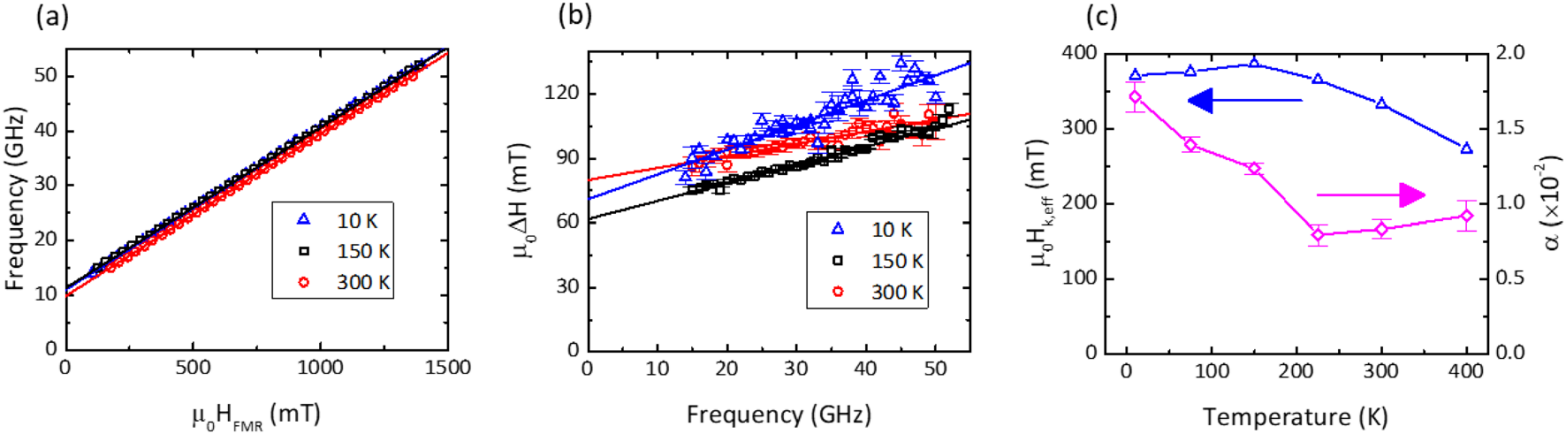
SAF B, temperature dependent measurements of (**a**) *H*_res_ vs. *f*; (**b**) ∆*H* vs. *f*; and (**c**) *H*_eff_ and α versus temperature.

The dynamic properties of the intermediate IEC sample, SAF B, are summarized in Fig. 4. The higher IEC field in this sample did not permit for observation of the optic precession mode in this sample. Figure 4a highlights the frequency versus resonance field trends at 20 K, 300 K and 400 K. The linear trends with overlaid fitting data were used to extract the effective anisotropy and the *g-*factor for this sample. The *g*-factor remained flat over this sample series to within 1%. Figure 4b shows the linewidth versus frequency at the same temperatures. While the temperature dependent changes in the effective anisotropy and *g-*factor are difficult to discern in Fig. 4a, there were more substantial changes in the slope of the trendlines in Fig. 4b, reflecting changes in the Gilbert damping and the ILB. Unlike the SAF A, we note that the effective anisotropy of SAF B increased from 0.28 T to 0.37 T with decreasing temperature, consistent with the proportionality of magnetic anisotropy with the saturation magnetization, which similarly shows increases over the same range. The Gilbert damping is nearly insensitive to temperature from 300 K down to approximately 150 K, after which it continuously rises from 0.008 to 0.017 at 10 K. The combination of interlayer exchange coupling, an anisotropy field of approximately 0.3 T and Gilbert damping in a 12 nm thick FePd SAF is of significant technological merit for memory devices, where the estimated magnetic anisotropy barrier of a 10 nm diameter nanopillar formed from this film would have an energy barrier $\Delta$ greater than 200 *k*_B_*T* at room temperature, and nearly 120 *k*_B_*T* at 265 °C, the solder reflow temperature during integrated circuit packaging.

### Temperature dependence of the Gilbert damping and perpendicular anisotropy of a high IEC pSAF and the spin-flip transition

**Figure 5:**
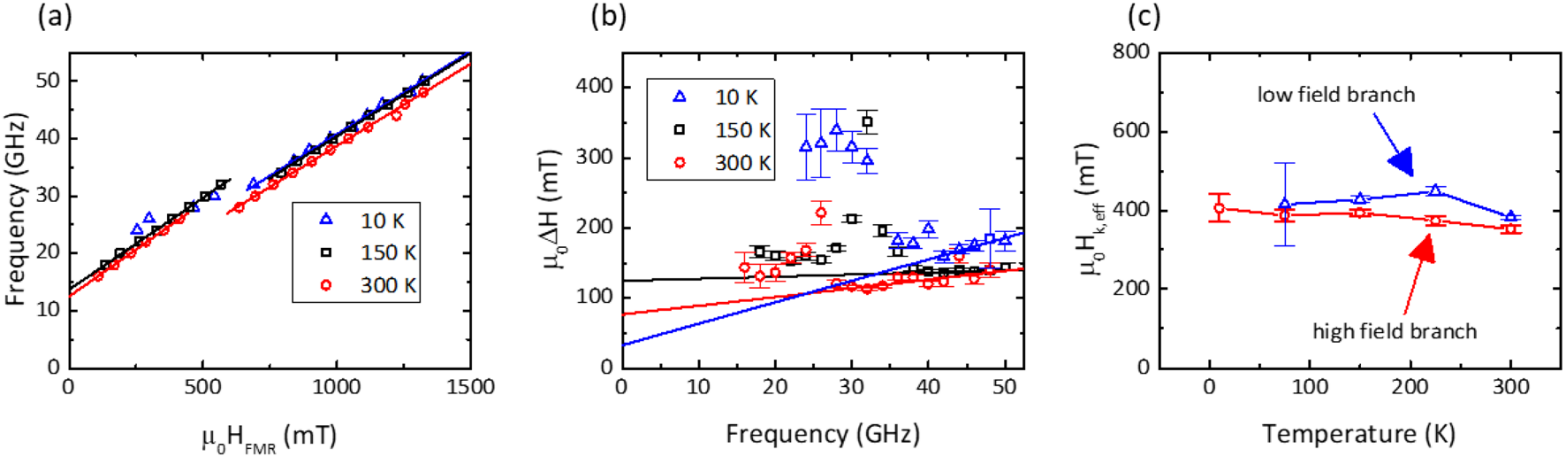
SAF C, temperature dependent measurements of (**a**) *H*_res_ vs. *f*; (**b**) ∆*H* vs. *f*; and (**c**) *H*_eff_ versus temperature for the low field branch and high field branch, respectively.

We now proceed to the highest interlayer exchange sample, SAF C. In Fig. 1b we showed that this sample had a reorientation of the two FePd layers at approximately 0.6 T, which has certain impact on the ferromagnetic resonance behavior. Figure 5a shows example resonance frequency versus out-of-plane field data at 300 K, 150 K and 10 K. Common to all examined temperatures, the spectra were discontinuous around the IEC field, as the reorientation of the two SAF layers led to a reduction in the effective anisotropy field by a moderate quantity of approximately 30 mT associated with the coupling between the two layers. A moderate increase in the effective anisotropy field is seen with decreasing temperature for the high field data generated with the SAF saturated into a parallel orientation, consistent with the previous samples.

The linewidth versus frequency data similarly showed two trends for SAF C. We observe relatively larger linewidths when the two FePd layers are anti-parallel and for fields much lower than the IEC field. As the exchange field is approached, the rotation of the two FePd layers into alignment (which we define as the spin-flip transition) is associated with significant increase in the linewidth, reaching a maximum around the IEC field. It is likely that the non-collinear magnetizations of the two layers leads to additional line broadening, an effect previously seen in other multilayered magnetic films^45^. At sufficiently large external fields, the mutual alignment of the two FePd layers leads to a reduction in the linewidth and restoration of the linear trend of linewidth versus frequency, as shown in Fig. 5b. To evaluate the Gilbert damping of this SAF, we estimate the linear trend of the linewidth versus frequency at these higher frequencies as can be seen by the overlaid lines in Fig. 5b. In this high IEC-optimized SAF, we find an IEC field of 0.6 T, an anisotropy field of 0.38 T and a Gilbert damping of 0.02.

## Discussion

The FePd-based synthetic antiferromagnet is a versatile building block for engineering large perpendicular magnetic anisotropy, variable interlayer exchange coupling and low Gilbert damping. As an epitaxial SAF, this structure has several technologically-ready properties, most notably the stability of large IEC following high temperature (500 °C) thermal processing and sufficiently high perpendicular magnetic anisotropy energy density for an array of 10 nm diameter nanomagnet devices to prevent data loss during solder reflow (265 °C) processing. Because the perpendicular magnetic anisotropy comes from the crystal lattice, it is possible to engineer nanomagnets with an aspect ratio of nearly unity, taking advantage of the low shape anisotropy cost and reducing the additional fabrication burden of the lithographic patterning of high aspect ratio nanomagnets, currently envisioned for future sub-10 nm magnetic memory applications. One significant obstacle may be producing FePd with these properties on technologically relevant surfaces. Future efforts should be directed toward identifying classes of materials, particularly those that prefer to form < 100 > columnar grains when grown above amorphous or polycrystalline surfaces at the interface to back-end-of-line process flows. Such materials could replace the bulk MgO(001) substrate used to seed the epitaxial Cr/Pt/FePd/Ir/FePd stacking structure, key to the high PMA, low damping and interlayer exchange. Nevertheless, the current results should encourage further advances in magnetic materials for advanced magnetic memory technologies.

## Methods

Single-crystalline MgO(001) substrate crystals (MTI Corporation^46^) of 0.5 mm thickness were loaded into a custom, multi-target, ultrahigh vacuum magnetron deposition facility with a base pressure lower than 6.7 × 10^–8^ Pa (5 × 10^–10^ Torr) in a residual H_2_ atmosphere as determined by an *in-situ* residual gas analyzer. Substrates were loaded immediately after removal from individual vacuum-sealed packaging and without any *ex-situ* cleaning. Surface preparation before deposition was accomplished by a 90 min vacuum heat-treatment *in-situ* on a heated deposition stage at 600 °C, at a vacuum lower than 6.7 × 10^–6^ Pa (5 × 10^–8^ Torr). A layer stacking structure of Cr(15)/Pt(4)/FePd/Ir/FePd was grown at a deposition temperature of 350 °C, while a capping bilayer complex of Ru(2)/Ta(3) was grown at room temperature. Layers were grown in a 3 mTorr Ar atmosphere under continuous azimuthal rotation of 20 revolutions per minute. The deposition rates were monitored by *in-situ* quartz crystal monitors that were calibrated by *ex-situ* measurements of thicker calibration films of each material. The deposition rates for each material were (in nm/s): Cr(0.033); Pt(0.13); FePd(0.029); Ir(0.017); Ru(0.020) and Ta(0.033). Three SAF structures were explored here: FePd(4)|Ir(1.3)|FePd(5); FePd(4)|Ir(0.9)|FePd(5) and FePd(4)|Ir(0.8)|FePd(2.5). The entire structure was *in-situ* annealed at 500 °C in vacuum immediately following capping layer deposition.

Magnetization versus applied magnetic field measurements were carried out in a vibrating sample magnetometer at ambient temperature.

The ferromagnetic resonance measurements performed on the SAF structures used a coplanar waveguide setup with modulation of the applied magnetic field for lock-in detection of the microwave transmission. The frequency of the microwave excitation was held fixed while the applied magnetic field (perpendicular to the plane of the SAF) was swept through the resonance condition. Microwave frequencies up to 50 GHz were used. The applied magnetic field was modulated with a sinusoidal ac field having frequency 220 Hz and amplitude of $\approx$ 0.1 mT to 1 mT, which was referenced to a lock-in amplifier. The transmitted microwave power was rectified using a Schottky diode with the output connected to the lock-amplifier, which measured the differential absorption. The measurements were performed in a Quantum Design Physical Property Measurement System using a probe designed by NanOsc.

Specimens for transmission electron microscopy were prepared by mechanical lapping and polishing followed by argon ion milling to electron transparency. They were imaged using an FEI Titan 80–300 operated at 300 kV and utilizing a high-angle annular dark field detector in scanning mode to acquire Z-contrast images.

### Supplementary Information

Supplementary Information.

## Data Availability

The datasets generated during and/or analyzed during the current study are available from the corresponding author on reasonable request.
