# Supplementary material for: Low Gilbert damping and high perpendicular magnetic anisotropy in an Ir-coupled L10-FePd-based synthetic antiferromagnet
Source: Sci Rep. 2024 Jun 10;14:13290. doi: 10.1038/s41598-024-63475-0 (PMC11164879; doi:10.1038/s41598-024-63475-0)
Supplement: Supplementary file 1 — Supplementary Information. [file 41598_2024_63475_MOESM1_ESM.docx]

**Supplementary Information for
Low Gilbert damping and high perpendicular magnetic anisotropy in an Ir-coupled L1_0_-FePd-based synthetic antiferromagnet**

**W. K. Peria,^1^ M. B. Katz,^2^ J.-P. Wang,^3^ P. A. Crowell,^1^ and D. B. Gopman^2,*^**

^1^School of Physics and Astronomy, University of Minnesota, Minneapolis, MN, 55455, USA

^2^Materials Science and Engineering Division, NIST, Gaithersburg, MD, 20899, USA

^3^Department of Electrical and Computer Engineering, University of Minnesota, Minneapolis, MN, 55455, USA

^*^daniel.gopman@nist.gov

**Supplemental X-ray diffraction**

We have carried out symmetrical x-ray diffraction (XRD) in a parallel beam configuration to illustrate the (1) preferred (001) growth direction out-of-plane for each of the layers (Pt, FePd) on the single-crystal MgO(001)-cut substrate by the host of (002) reflections, and the appearance of the otherwise forbidden (001) reflection due to the superlattice ordering within the FePd layer. Supplemental Figures S1(a)-(c) illustrate the XRD intensity versus two-theta/theta scans for SAF samples A, B and C. The ratio of integral intensity between the (002) and (001) referenced FePd peaks is proportional to the degree of L10 ordering which can be estimated by dividing by the theoretical intensity ratio. A moderate degree of L1_0_ ordering is observed in all three samples, with an estimated order parameter *S* of 0.54 ± 0.07, 0.58 ± 0.06 and 0.64 ± 0.07 for the samples SAF A, SAF B and SAF C, respectively.

Supplementary Figure S1: Symmetric x-ray diffraction in parallel beam configuration showing Intensity versus Two-theta for samples (a) SAF A; (b) SAF B and (c) SAF C.

(c)

(b)

(a)


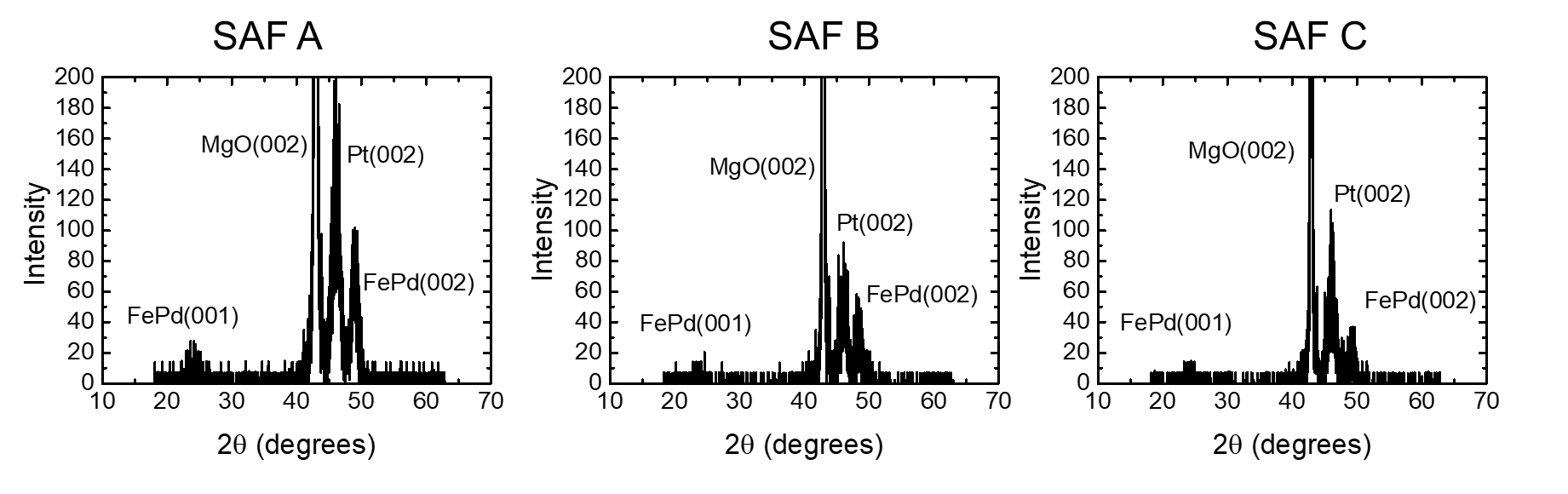


**Supplemental TEM micrographs and intensity profiling**

The HAADF-STEM images from Figure 2 are reproduced here along with line profiles of the contrast intensity. Due to the chemical contrast in STEM, the sample thicknesses can be estimated here. Additionally, an oscillatory contrast can be seen within the FePd layers for each sample, associated with the double contrast originating from *Z* contrast within the ordered Fe and Pd planes.


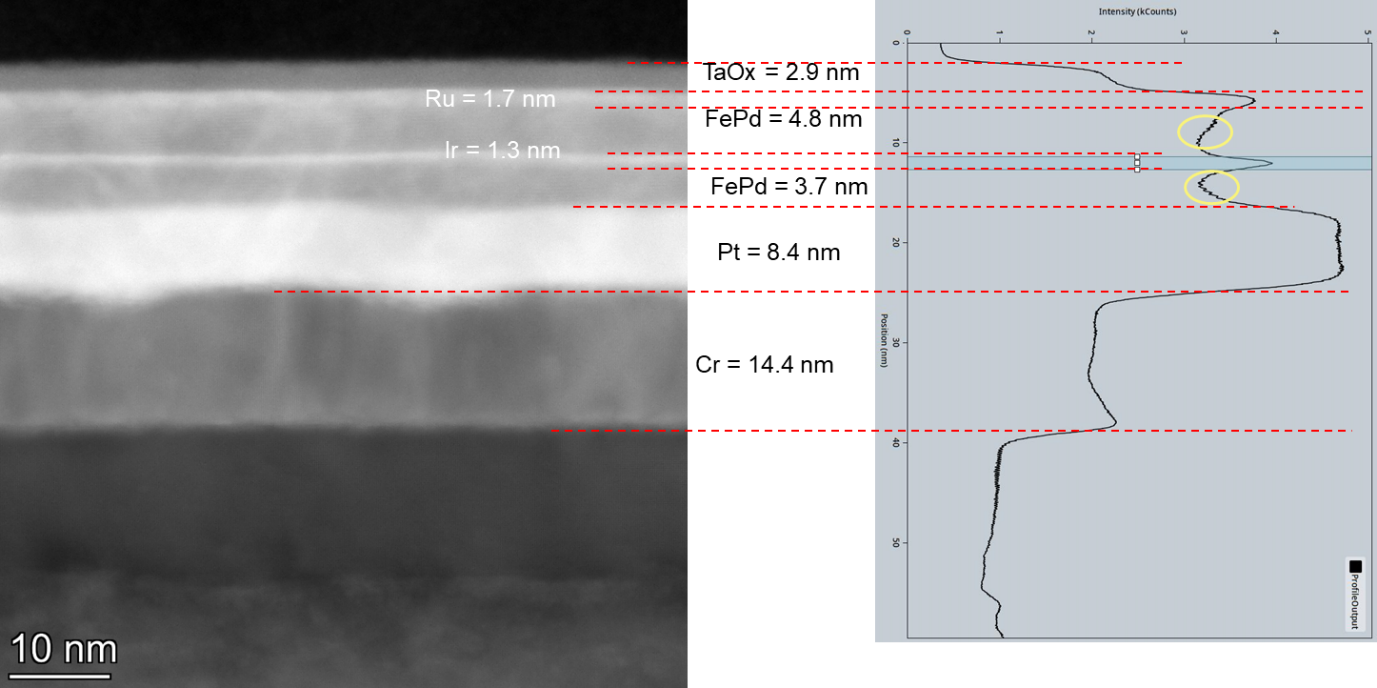


Supplementary Figure S2: (left) HAADF-STEM image of SAF A and (right) associated intensity depth profile


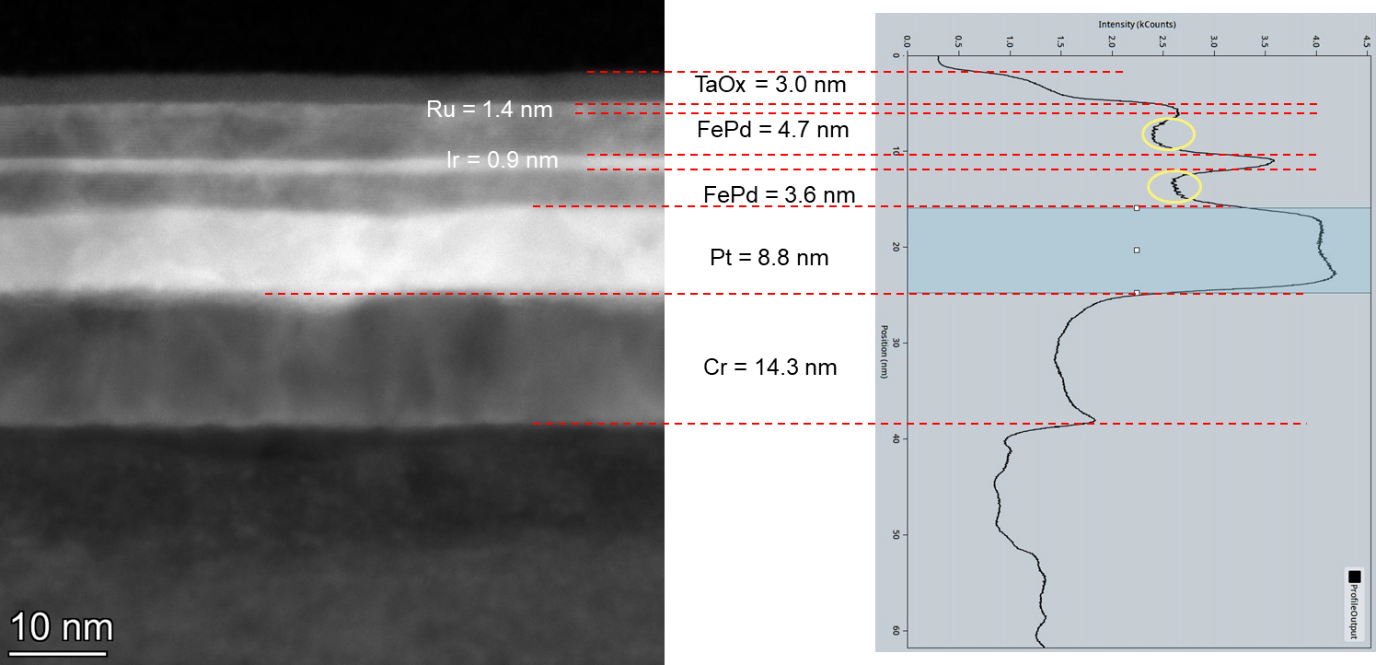


Supplementary Figure S3: (left) HAADF-STEM image of SAF B and (right) associated intensity depth profile


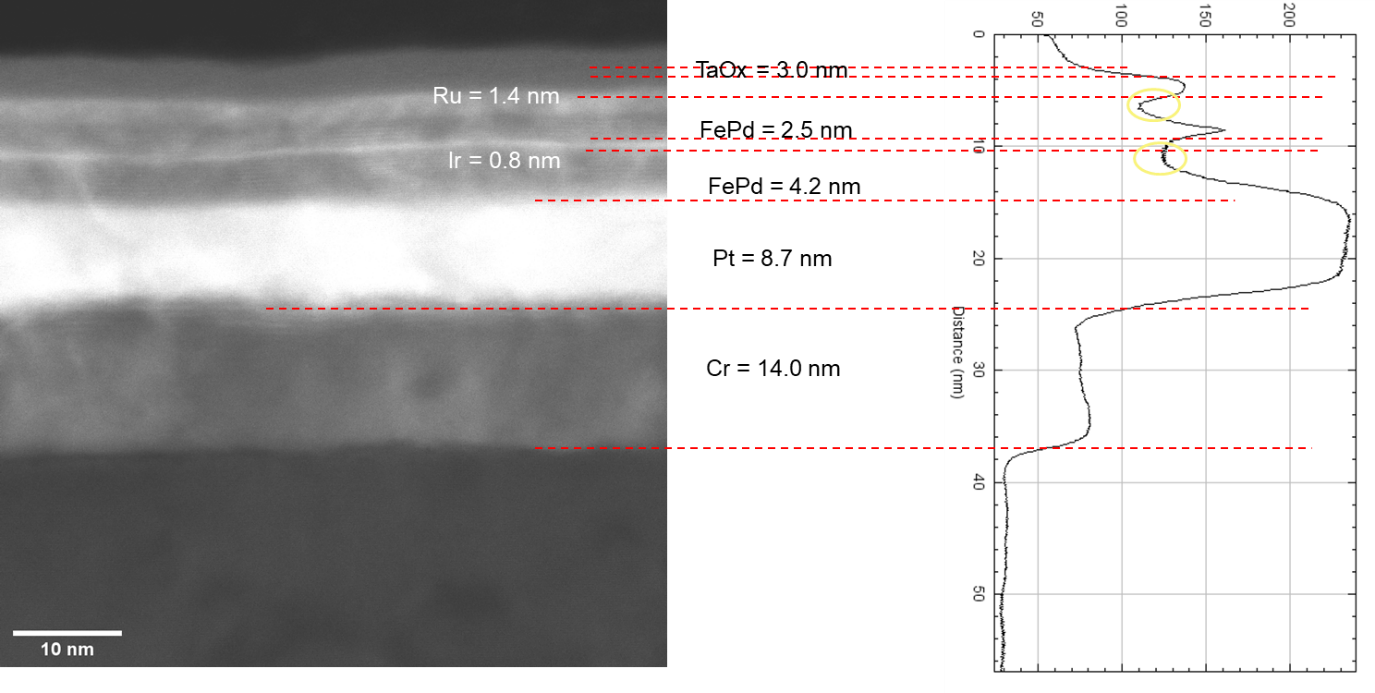


Supplementary Figure S4: (left) HAADF-STEM image of SAF C and (right) associated intensity depth profile
